# Peroxiredoxins - Urinary Surveillance Biomarkers in Urothelial Cancer

**DOI:** 10.7150/jca.69811

**Published:** 2022-06-13

**Authors:** Nitu Kumari, Pawan Vasudeva, Pranay Tanwar, Showket Hussain, Anup Kumar, Usha Agrawal

**Affiliations:** 1 ICMR-National Institute of Pathology, New Delhi, Safdarjung Hospital 110029, India.; 2 Department of Urology, VMMC, Safdarjung Hospital 110029, India.; 3 Dept of Laboratory Oncology, Dr. BRA-Institute Rotary Cancer Hospital, AIIMS, New Delhi, India.; 4 Division of Cellular & Molecular Diagnostics, ICMR-National Institute of Cancer Prevention & Research, NOIDA, India.

**Keywords:** Bladder cancer, Urinary PRDX1, and PRDX2

## Abstract

**Introduction:** Urinary bladder cancer ranks the fourth most common cancer in men worldwide. Peroxiredoxins (PRDXs) are antioxidant enzymes that play an important role in cell proliferation and apoptosis. In the present study, we investigated whether PRDX 1 and 2 can be used as a urinary biomarker for surveillance of recurrence in urothelial cancer.

**Materials and Methods:** PRDX1 and PRDX2 expression levels were examined in 119 bladder tumor specimens by immunohistochemistry and in 150 urine samples (case: 100; healthy controls: 50) using ELISA and their association with recurrence and survival of patients was evaluated.

**Results:** Immunohistochemistry on FFPE tissue showed that both PRDX1 and PRDX2 were positive in bladder tumors and expressed in the cytoplasm and membrane of tumor cells. A significant elevation of urinary PRDX1 and PRDX2 concentration was found in bladder cancer patients and recurrent cases compared to the urine of healthy controls and primary bladder cancer patients (p<0.001 & p<0.01) respectively. However, the concentration of both proteins was not found associated with survival.

**Conclusion:** Elevated urinary PRDX1 and PRDX2 in bladder cancer patients was found to be associated with recurrence and the estimation of urinary PRDX1 and PRDX2 during follow-up may help to extend the period between cystoscopies in patient follow-up.

## Introduction

Urothelial carcinoma is the most prevalent genitourinary male cancer with the highest morbidity rate 1 Despite treatment, 70% of bladder tumors recur 2 and only lifelong cystoscopy helps in surveillance. The secreted or excreted byproduct of the tumor can be used as diagnostic or prognostic markers, and modify clinical management and follow-up. Numerous studies have assessed urinary biomarkers to detect possible biomarkers that could be predictive of recurrence 3-7. Various molecular factors of cell proliferation, redox regulation, cell differentiation, etc. may promote tumor resistance against therapy and could be responsible for frequent recurrence of the tumor 8, 9.

Peroxiredoxins (PRDXs) are non-selenium-dependent glutathione peroxidases, 22 to 27 kDa, and scavenge peroxides, organic hydroperoxides, and peroxynitrite. They are ubiquitously expressed, thiol-dependent peroxidases, with a conserved cysteine residue. The most common six isoforms of PRDX (PRDX 1-6) are found in mammals and associated with proliferation, apoptosis and differentiation 10,11. PRDXs have a cytoprotective antioxidant function and play a role in cellular processes involving redox homeostasis. The high expression of PRDX2 is reported to be associated with increased resistance to chemotherapeutic drugs and associated with a high proliferation rate and tumor recurrence 12,13. Hence, alteration in expression of PRDXs in disease appears important to evaluate PRDXs as diagnostic or surveillance biomarkers. Therefore, in the present study, the expression levels of PRDX 1 and PRDX2 were evaluated in bladder cancer tissue and patients' urine, and their association with recurrence on follow-up was evaluated.

## Material and Methods

### Sample collection

This study included patients with bladder cancer presenting to the Outpatient Department of Urology, Safdarjung Hospital, New Delhi for four years. Samples used for this study were obtained with informed consent and with the approval of the Safdarjung hospital Ethics Committee (EC/SJH/VMMC/Project/I4/07-325). The patients who presented with hematuria to urology OPD were examined by cystoscopy and urine cytology. The patients who were positive for malignancy on urine cytology with cystoscopic lesion confirmed histopathologically were included in the study and samples were collected after written consent was obtained. Patients with histological grades pTa, metastatic disease, concurrent tumours and associated upper tract transitional cell carcinoma were excluded from the study. Controls included age and sex-matched healthy volunteers who did not have any other history of co-morbidity or fever in the 3 weeks before sample collection.

A total of 274 samples (119 tumor tissue, 5 normal mucosa and urine samples from 100 bladder cancer patients and 50 healthy controls) were included in this study. Demographic details of samples used are summarized in Table 1.

### Immunohistochemistry

The slides with FFPE sections were placed in an incubator at 55 °C for 15 to 20 minutes. Slides were rehydrated and heat-induced antigen retrieval was performed in the TE buffer (Tris-EDTA buffer) in a water bath at 95 °C for 20 minutes. Endoperoxidase activity was blocked with 3% H_2_O_2_ and slides were incubated with primary antibody (PRDXI- 1:100 & PRDXII- 1:200; Thermo Scientific) at 4 °C overnight. Slides were incubated with HRP conjugated secondary antibody the next day. DAB was used as a chromogen, and sections were counter-stained with Haematoxylin. Slides were mounted with DPX and observed under the light microscope.

### Urine ELISA (Enzyme-linked immunosorbent assay)

Collected urine samples were centrifuged at 4,000g for 5 min and the supernatant was collected for use. Enzyme-linked immunosorbent assay (ELISA) was used to quantify PRDX1 (Abcam, ab185983) and PRDX2 (R&D Systems, DY3489) concentration in urine. The assays were performed in duplicate. The protocol given in the manufacturers' instructions was followed and readings were obtained at 450nm.

### Statistical analysis

A Chi-square test was performed to find the significant association between categorical variables. The Mann-Whitney test was used to evaluate the significance of differences in marker concentration between each group. The median concentration of urinary markers was taken as a cut-off for survival analysis. Kaplan Meier analysis was performed for discerning the difference in recurrence-free survival and significance was computed by log-rank. A probability less than 0.05 was considered significant. All statistical analyses were performed using the statistical package for the Statistical Package for social sciences (SPSS) software version 19 (SPSS, Chicago, IL, USA).

## Results

### Expression of PRDX1 and PRDX2 in Bladder tumor

**Figure 1:**
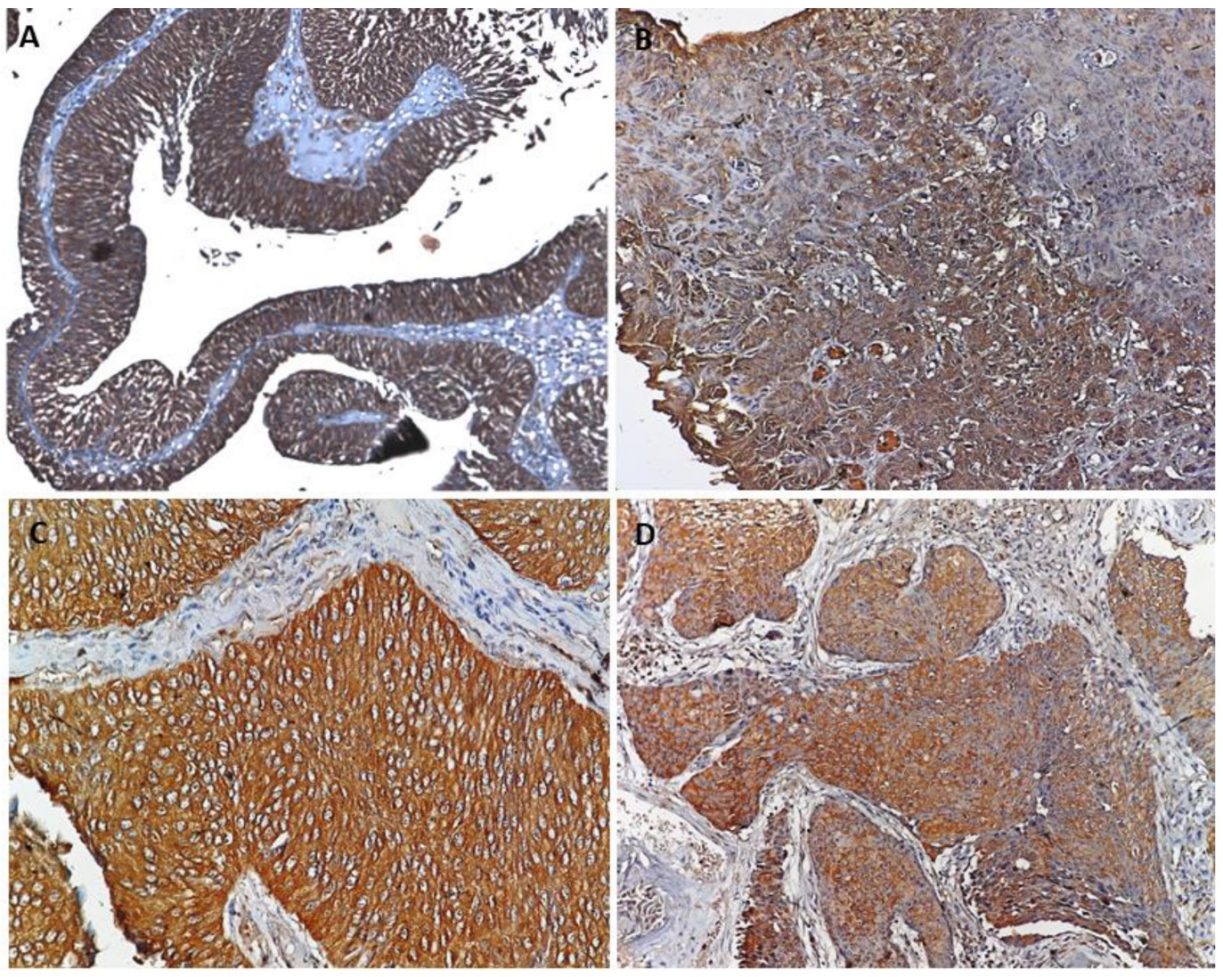
** A.** Representative image of PRDX1 and PRDX2 showed cytoplasmic and membranous expression in **(A & C)** non-invasive bladder cancer and **(B & D)** invasive bladder cancer.

Formalin-fixed, paraffin-embedded (FFPE) tissue from 119 cases of urothelial cancer and 5 cases with normal mucosa was used to evaluate the expression and localization of the protein (PRDX1 and PRDX2) in the tumor by immunohistochemistry. These cases included LGpT1 (n=53), LGpT2 (n=4), HGpT1 (n=41) and HGpT2 (n=21) for the expression and localization of markers. Hematoxylin and eosin staining were performed on each tissue section to confirm the presence of tumors in the section. PRDX1 and PRDX2 were both found to be expressed in tumor cells and these proteins were absent in normal mucosa evaluated as control. Both proteins were expressed in cytoplasm and membrane in all subgroups of bladder tumors. The statistical test showed no significant difference in expression of the proteins between the grades and stage of the tumor (Figure 1).

### Estimation of PRDX1 and PRDX2 in urine

**Figure 2:**
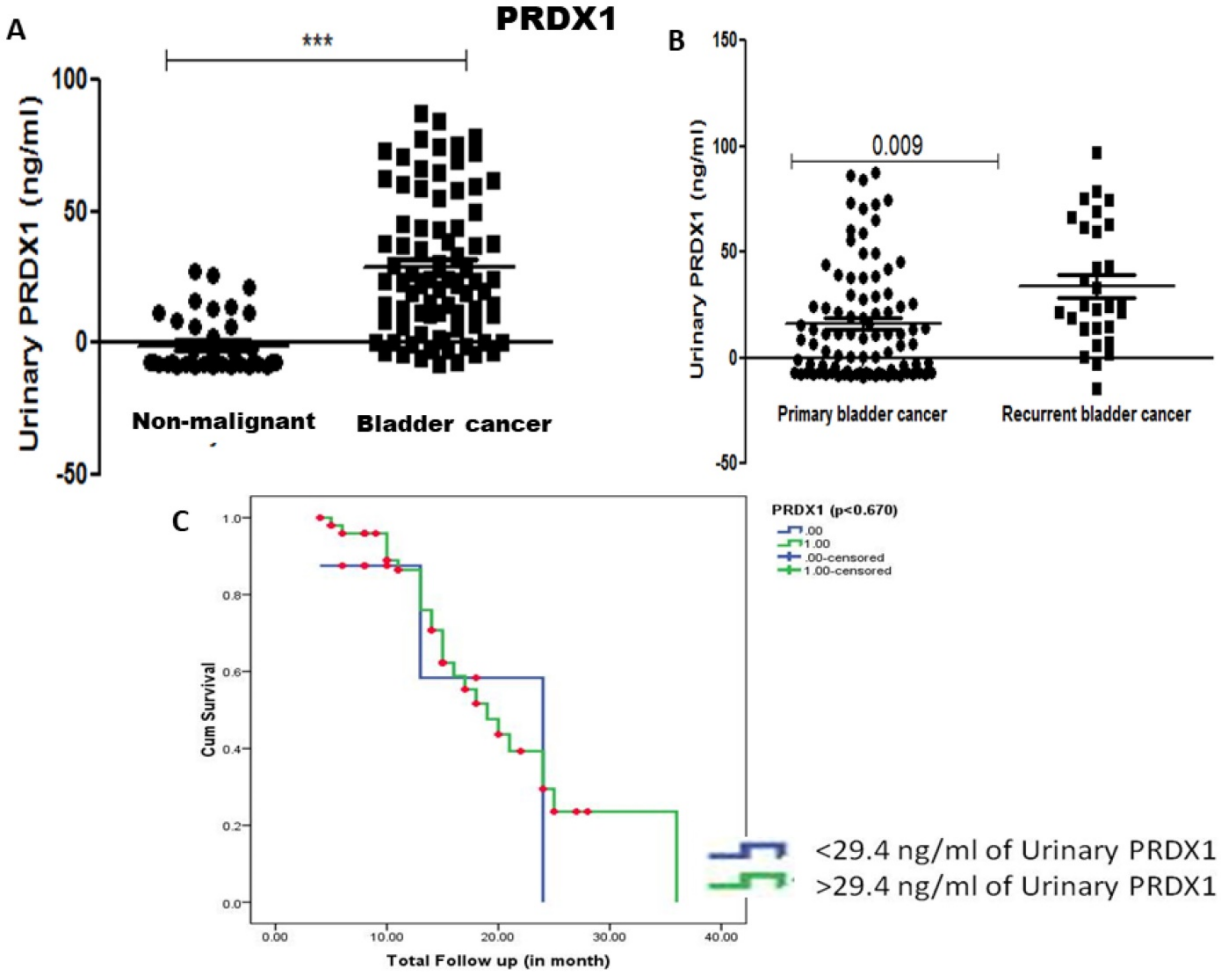
** A)** Urinary concentration of PRDX1 was estimated in the urine sample and found significantly elevated in the urine of bladder cancer patient compared to non-malignant control (p-value < 0.001, calculated by Mann Whitney U test). **B)** Elevated urinary concentration of PRDX1 was found in the urine sample of recurrent bladder cancer patient compared to primary bladder cancer and **C)** Kaplan Meier analysis showed the concentration of PRDX1 (29.4 ng/ml) was not associated with recurrence-free survival (p=0.67).

Levels of PRDX1 and PRDX2 protein were estimated in the urine of 100 cancer patients and 50 non-malignant controls by ELISA. The median concentration of urinary PRDX1 was 29.4 ng/ml and significant elevation of urinary PRDX1 was found in bladder cancer patients compared to urine from healthy controls (p<0.001). Though the median concentration of urinary PRDX1 was higher in the urine sample of recurrent bladder cancer patients compared to primary bladder cancer patients the difference was not statistically significant (Figure 2). Kaplan Meier survival analysis with median concentration (29.4 ng/ml) of urinary PRDX1 as cut-off value showed that the concentration of urinary PRDX1 was not associated with recurrence-free survival (Figure 2).

**Figure 3:**
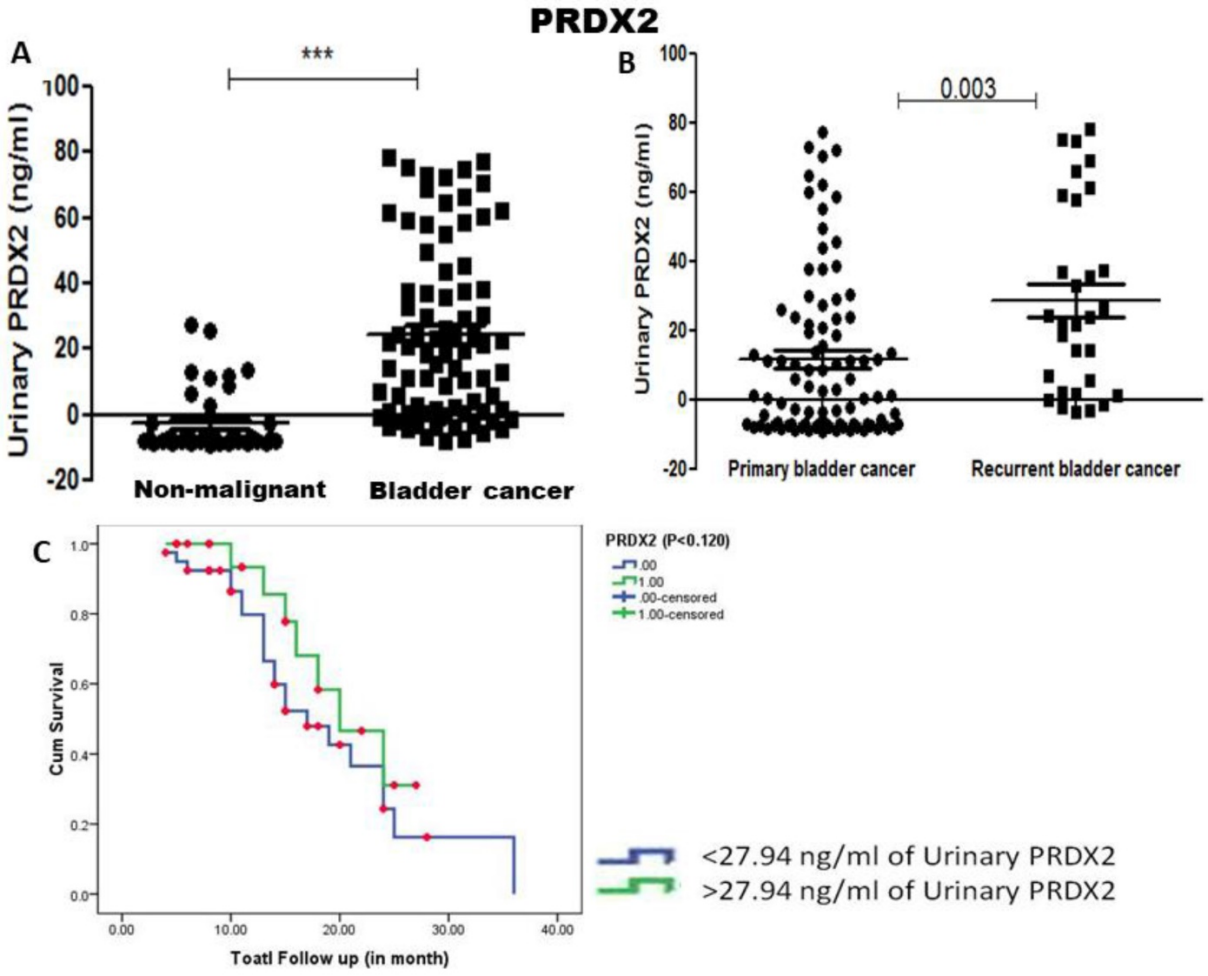
** A)** Urinary concentration of PRDX2 was found significantly elevated in bladder cancer patients compared to non-malignant control (p-value < 0.001, calculated by Mann Whitney U test). **B)** Significant elevation in the concentration of urinary PRDX2 in recurrent bladder cancer compared to primary bladder cancer (p-value=0.003) and **C)** Kaplan Meier analysis showed lower concentration (<27.94 ng/ml) of PRDX2 associated with recurrence and poorer survival of bladder cancer patients (log-rank t-test, p=0.125).

Urinary PRDX2 concentration was similarly found to be significantly more in bladder cancer patients compared to urine from healthy controls (p<0.001). Unlike PRDX1, significant elevation of urinary PRDX2 was found in recurrent bladder cancer patients compared to primary bladder cancer patients (p=0.003) (Figure 3). Median concentration 27.94 ng/ml of urinary PRDX2 was taken as cut-off value and Kaplan Meier survival analysis showed no association with recurrence-free survival (p=0.125) (Figure 3).

PRDX1 and 2 both act as diagnostic markers, but it is PRDX2 which is significantly increased in recurrence and can be used as a urinary surveillance marker in bladder cancer patients.

## Discussion

Oxidative stress has been shown to be associated with prognosis in urinary bladder cancer 14. Oxidative stress stimulates the expression of PRDXs and is regulated by transcriptional mechanisms 15. Peroxiredoxins (PRDXs) are a member of the glutathione peroxidases family which destroys peroxides, organic hydroperoxides, and peroxynitrite 16. Peroxiredoxin 1 (PRDX1) is an antioxidant enzyme and plays an important role in H_2_O_2_-mediated cell signaling 17. PRDX1 inhibits the activation of oncogenes (c-Abl and c-myc, and PTEN) which is essential for its tumor-suppressive function 18. Both PRDX 1&2 protect mitochondria and affect growth and differentiation by scavenging hydrogen peroxide in the mitochondria 19. These proteins are overexpressed in malignancy 20,21,22 and may be a potential target for cancer therapy.

We found expression of PRDX1 was significantly increased in bladder cancer tumors when compared to normal mucosa and similar results are reported in esophagus squamous cell carcinoma 23,24 and colorectal carcinoma 25. Urinary concentration of both PRDXs was found significantly elevated in patients but was not associated with the recurrence-free survival of the patient.

A similar study by Quanet et al. found that enhanced PRDX1 expression in bladder cancer tissue is strongly associated with development and progression but its expression did not correlate with disease-free survival in patients with bladder cancer 26. Gao et al performed an analysis of the expression of peroxiredoxins across 33 different organ cancers from TCGA database and found that PRDX1 was associated with poor survival and PRDX2 with favorable survival 27 Overexpression of PRDX1 was found to be an independent poor prognostic factor for overall survival in hepatocarcinoma and the role of SUMO in carcinogenesis has been demonstrated 28. Soini et al demonstrated that tissue expression of peroxiredoxins did not have an association with prognosis but that serum and urine concentration of 8OHdG, another oxidative enzyme, did have an association. They also suggested that elevated levels of PRDXs may be used as a target for therapy 14. Our study did not show statistically significant association of either marker with survival but were both significantly increased in patients compared to controls and may function as surveillance markers.

The study is limited by the sample size. A larger cohort and ROC analysis would have given a more accurate cut-off. Literature review showed studies which demonstrated tissue expression of peroxiredoxins and association with survival was analyzed, but the evaluation of these as disease status markers in non-invasive samples has not been performed. The significantly elevated urinary concentrations of PRDX1 & 2 in bladder cancer patients compared to healthy individuals suggests that these are potential biomarkers and can be used for disease surveillance.

## Conclusion

The high expression of both markers in tumour tissue, irrespective of grade and muscle invasion as well as their elevated concentrations in urine of recurrent cases makes these two PRDXs good urinary markers for follow-up of bladder cancer cases.

## Figures and Tables

**Table 1:** Demographic details of study cohort

	Immunohistochemistry		ELISA	
	Patients, n=119 (%)	Normal mucosa, n=5 (%)	Patients, n=100 (%)	Control, n=50 (%)
Median Age	58	54	58	55
1st to 3^rd^ IQR	53 to 68	51 to 60	49 to 56	45 to 61
**Gender**				
Female	16 (14)	0	14 (14)	12 (24)
Male	103 (86)	5 (100)	86 (86)	38 (76)
**Grade**				
LG	57 (48)	--	--	--
HG	62 (52)	--	--	--
**Stage**				
PT1	94 (78)	--	--	--
PT2	25 (22)	--	--	--
Recurrence	--	--	41 (27)	--

IQR, interquartile range; F, Female; M, Male; LG, low grade; HG, High grade; pT1, Non-muscle invasive; pT2, Muscle invasive.
